# The impact of a national transition of treatment platform on the acceptance of internet-delivered cognitive behavioral therapy: A longitudinal study among Norwegian therapists

**DOI:** 10.1016/j.invent.2026.100965

**Published:** 2026-06-13

**Authors:** Reidar Nævdal, Christiaan Vis, Inger Lise Teig, Derek de Beurs, Robin Maria Francisca Kenter

**Affiliations:** a Research Centre for Digital Mental Health Services, Division of Psychiatry, Haukeland University Hospital, Post Office Box 1400, Bergen, N-5021, Norway; b University of Bergen, Department of Global Public Health and Primary Care, Post Office Box 7804, Bergen, N-5020, Norway; c Amsterdam University Medical Centre, Department of Public and Occupational Health and Amsterdam Public Health Research Institute, Amsterdam, Netherlands; d University of Amsterdam, Department of Clinical Psychology, Amsterdam, Netherlands

**Keywords:** Internet-delivered cognitive behavioral therapy, Technology Acceptance, TAM, Implementation, Digital mental health, Digital therapist

## Abstract

**Background:**

Therapists' acceptance of internet-delivered cognitive behavioral therapy (iCBT) is critical for successful and sustained implementation. However, little is known about how acceptance is affected by changes in delivery platforms.

**Objective:**

To investigate how therapists' acceptance of iCBT changes during a national transition to a new delivery platform.

**Methods:**

During a national change in delivery platform, 46 therapists (69% of the national population; N = 67) providing iCBT in Norwegian specialist health services, completed questionnaires at five time points over 18 months. The study applied an extended Technology Acceptance Model (TAM) which accounted for both patients and therapists as users of iCBT. Measures included TAM constructs, individual characteristics and place of employment. Multilevel models assessed changes in acceptance over time and baseline variance at therapist and organizational levels.

**Results:**

Overall acceptance of iCBT was high at baseline and remained largely stable throughout the transition. Therapist perception of patients' ease of use increased significantly over time and was clearly distinguished from their perception of their own user experience. Notably, therapists' acceptance of the new platform was high even before they had gained hands-on user experience.

**Conclusion:**

Among experienced iCBT therapists', acceptance appears resilient to disruptions caused by changes in treatment platforms. Acceptance of a new platform is not dependent on positive user experience alone and may be affected by efforts that influence therapists' expectations before implementation. Therapists' perceptions of their patients' user experience are likely important for their acceptance but remain poorly understood.

## Highlights

• Therapists' acceptance of iCBT was assessed during a national platform transition.
• Acceptance of iCBT was high at baseline and remained stable during the transition.
• Therapists perceived use to become easier for patients after the platform change.
• Therapists did not perceive any change in their own ease of use or usefulness.
• Therapists had high acceptance of the new platform before using it.

## 1 Introduction

Therapist-guided internet-delivered cognitive behavioral therapy (iCBT) is as effective as face-to-face therapy for depression and anxiety disorders (Carlbring et al., 2018; Etzelmueller et al., 2020; Hedman-Lagerlöf et al., 2023). It holds the promise of improving access to care (Ebert et al., 2018; Stein et al., 2022) and contributes to equitable health care by reducing a global treatment gap (Alonso et al., 2018; Moitra et al., 2022). Although it can be considered both practical and acceptable for end-users (Andrews et al., 2018), the implementation and dissemination of iCBT at scale remains a challenge (Duffy et al., 2025; Folker et al., 2018; Vis et al., 2018).

A defining feature of iCBT is the delivery method (Andersson et al., 2016). While the treatment content patients receive is usually the same as established cognitive behavioral therapy (CBT) delivered face-to-face, iCBTs use a *treatment platform* to deliver this content via the internet (Andersson et al., 2016). Treatment platforms are information technology systems (IT systems) used to host and deliver the treatment content. These online software systems can vary in both functionality and user interface. This means that while two iCBT treatments may contain the same therapy, differences in treatment platform can affect the user experience of both the patients and the therapists. Differences in functionality and user interface may, in turn, influence important metrics such as user engagement, and affect which information users are exposed to when interacting with the platform.

Switching the platform used to deliver iCBT is a common occurrence. For example, Norway's first three iCBT programs were initially developed, tested, and delivered via a Swedish treatment platform before being integrated into a new platform for Norway (Nordgreen et al., 2019; Nordgreen et al., 2018a, Nordgreen et al., 2018b). There are also several instances where a platform that has been used over time is updated or replaced. Such changes may be driven by legal requirements to comply with regulations on user interface, data security, or public procurement, or they may aim to improve functionality and enhance usability.

### 1.1 Consequences of changing information technologies

Updating the IT infrastructure, or introducing a new IT system, often disrupts work processes, leading to user frustration and workarounds (Tsai et al., 2020; Zheng et al., 2020). While the new technology may have improved functionality or user interface, adoption and actual use of this technology are not just a consequence of its functionality. Instead, studies show that non-technological factors like organizational support, implementation processes, and social dynamics in the organization are important determinants of user adoption (Huang et al., 2020; Miake-Lye et al., 2023). These non-technological factors are commonly conceptualized in models such as the Technology Acceptance Model (TAM) (Davis, 1989; Davis et al., 2024). The construct of *acceptance* refers to the degree to which a user is willing to employ a technology for intended purpose. The adoption and use of technology are, in other words, consequences of users' acceptance of any given IT system. Instead of reflecting the objective utility of the technology, acceptance depends on subjective perceptions of its usefulness, ease of use, prevailing social norms, and the availability of supportive structures (Venkatesh et al., 2003).

### 1.2 The dual-user nature of iCBT

An important characteristic of the technology used in iCBT is that it has two users, the patient and the therapist. Of the two, the therapist holds the responsibility of acting as gatekeeper to the treatment, tasked with informing the patient about treatment options, and delivering a treatment that is appropriate and suitable for the patient's needs. This makes their acceptance of iCBT particularly important for implementation and sustained use. While TAM is useful in understanding acceptance of single-user technologies, therapists' acceptance of iCBT may not be purely egocentric, but also reflect their perceptions of how useful or easy to use iCBT is for their patients. Although the dual-user characteristic of iCBT may shape therapists' acceptance of iCBT, this aspect remains underexplored in technology acceptance research. Furthermore, while changes or updates to the delivery platform will occur at some point for most sustained iCBTs, we know little about how such transitions affect established clinical practices. As gatekeepers to treatment, therapists delivering iCBTs play a significant role in the number of patients who will receive internet-delivered interventions. Understanding what affects their acceptance and how it evolves during technological transitions is therefore essential for supporting successful implementation and minimizing potential disruptions to care. By applying an extended TAM in a national, real-world platform transition, this study contributes novel longitudinal evidence on how therapists' acceptance of a dual-user technology evolves over time, including prior to hands-on experience with a new system.

### 1.3 Research question

The study included measures of therapists' perceptions of their patients' user experience in acceptance, and addressed the following research question: How does therapists' acceptance of iCBT change when they transition to a new treatment platform?

Since the new delivery platform would be unfamiliar to the therapists, they would need to gain experience and familiarity with a new user interface and functionality during the transition. In line with previous research on technology transitions (Tsai et al., 2020; Zheng et al., 2020), we therefore expected the platform change to cause an initial reduction in acceptance, regardless of whether the new platform was an objective improvement relative to the old platform. Over time however, we expected a potential recovery in acceptance levels as therapists gained experience and familiarity with the new platform.

## 2 Method

### 2.1 Study design

The study was a naturalistic, longitudinal study with repeated measures among Norwegian therapists delivering iCBT, conducted during a national transition from one iCBT platform to another.

### 2.2 Setting

The study was conducted within the Norwegian specialist health care services, where iCBT for panic disorder, social anxiety and depression are integrated treatment options (Nordgreen et al., 2018a, Nordgreen et al., 2018b, Nordgreen et al., 2019). Initially implemented in a single outpatient clinic, iCBT has been adopted by multiple clinics nationwide over the last decade, resulting in a decentralized organization with multiple distinct iCBT clinics, run by psychiatric hospitals or “hospital trusts”. While these clinics use the same iCBT treatments, the hospital trusts vary in location, catchment area, organization and culture. Although the use of iCBT has been expanded to cover most of Norway, little was known about therapist characteristics or how many there were at the time of data collection (Nævdal et al., 2025).

### 2.3 Platform change

In 2023, a national procurement process to select a new technology provider for the delivery platform of iCBT treatments was initiated. The goal of the procurement was to improve the platform's quality and add functionality (Sykehusinnkjøp HF, 2023). The procurement process was finalized in October 2023, and a transition period to the new platform began in April 2024. The platform change was done at the level of hospital trusts, with all iCBT clinics belonging to a trust transitioning at different times from the beginning of April 2024 to early March 2025.

### 2.4 Participants and data collection

All therapists delivering iCBT in the Norwegian specialist health care services were invited to participate in the study through an email containing information about the study and a unique link to a digital informed consent form and survey. In total, there were 67 eligible iCBT therapists working across 18 iCBT clinics. The clinics were in turn a part of 11 hospital trusts located in three of the four health regions in Norway. The recruitment process and detailed demographics of the therapists are described in Nævdal et al. (2025).

The data were collected using digital surveys with five repeated measures starting in February 2024, before the first hospital trust transitioned to the new platform (April 2024). The first four data collections (T0-T3) were done every third month, while the final data collection (T4) was done in September 2025, six months after the last hospital trust had transitioned.

### 2.5 Measures

#### 2.5.1 Conceptual framework

**Fig. 1:**
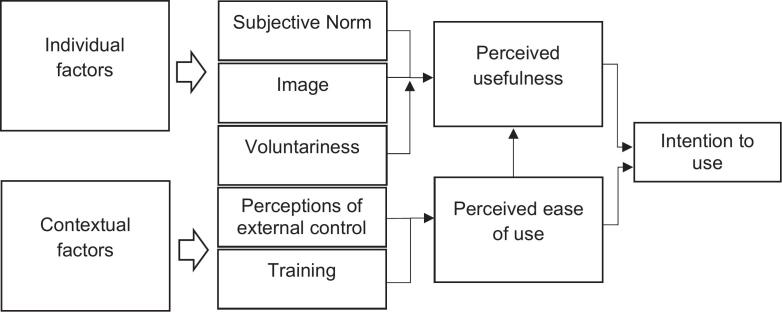
The technology acceptance model applied in the study.

To measure acceptance and how it was affected by the platform change, TAM was applied as a conceptual framework. To account for the dual-user nature of iCBT, the model was expanded to include therapists' perceptions of how iCBT is experienced by their patients as well as for themselves. The model was used to guide which variables to measure, and to interpret how they reflect the therapists' acceptance and their willingness to use iCBT (Fig. 1).

The TAM posits that a person's use of a technology is predicted by their *Behavioral Intention* (BI) to use the technology. BI is in turn predicted by the technology's *Perceived Usefulness* (PU) and its *Perceived Ease of Use* (PEOU) (Davis, 1989). A user's PU and PEOU are influenced by several latent variables (Venkatesh, 2000; Venkatesh and Bala, 2008; Venkatesh and Davis, 1996, Venkatesh and Davis, 2000), the user's context, and their personal characteristics.

#### 2.5.2 TAM variables

The variables in the applied TAM are presented in Table 1 along with their operationalization. These variables were used as outcome variables that measured the therapists' acceptance of iCBT, and included both the main measures of acceptance proposed by Davis (1989) (BI, PU and PEOU) and latent variables identified in subsequent research (Venkatesh, 2000; Venkatesh and Bala, 2008; Venkatesh and Davis, 1996, Venkatesh and Davis, 2000). To measure therapists' perceptions of how iCBT would be experienced by their patients, items measuring perceived usefulness (PU) and ease of use (PEOU) were rephrased to reflect the patients' perspective. Detailed descriptions of the translation and development of the scales are available in Nævdal et al. (2025), and an overview of original items and the adapted Norwegian items are available in Appendix A. Included variables were: Behavioral Intention to use Platform 1 (BI1), Behavioral Intention to use Platform 2 (BI2), Perceived Usefulness for themselves as therapists (TPU) and for their patients (PPU), Perceived Ease of Use for themselves as therapists (TPEOU) and for their patients (PPEOU), Subjective Norm (SNORM), Image (IMG), Voluntariness (VOL), Perceptions of External Control (PEC) and Training (TRN). BI1 and BI2 were introduced at T1 and were not available at baseline.

**Table 1:** Operationalization of TAM variables.

Variable	Operationalization
Behavioral intention to use Platform 1	The user's intention or likelihood to use Platform 1 in the future
Behavioral intention to use Platform 2	The user's intention or likelihood to use Platform 2 in the future
Perceived usefulness for themselves	The degree to which a user believes that using a particular technology will enhance their job performance or make tasks easier
Perceived usefulness for their patients	The extent to which a therapist believes that a particular technology will enhance or improve treatment for the patient
Perceived ease of use for themselves	the extent to which a user believes that using a particular technology will be free of effort
Perceived ease of use for their patients	The extent to which a therapist believes that a particular technology will be free of effort for the patient
Subjective norm	The degree to which an individual perceives that most people who are important to him/her think he/she should use the system
Image	The degree to which an individual perceives that use of an innovation will enhance his or her status in his or her social system
Voluntariness	The degree to which an individual believes they can choose whether to use the system as a treatment medium for patients they evaluate
Perceptions of External Control	The degree to which an individual believes that organizational and technical resources exist to support the use of the system
Training	The degree to which a user believes he has received sufficient training to operate the system in line with his or her responsibilities

#### 2.5.3 Internal consistency

Cronbach's alpha was used to assess internal consistency. At the first data collection (T0), values ranged from 0.44 to 0.94 across TAM scales (TPU = 0.81, PPU = 0.74, TPEOU = 0.80, PPEOU = 0.81, SNORM = 0.58, IMG = 0.86, VOL = 0.44, PEC = 0.73, TRN = 0.94). BI1 and BI2 showed good reliability at their first available timepoint (T1; α = 0.90 and 0.83, respectively).

#### 2.5.4 Contextual and individual characteristics

The main contextual variable was which hospital trust the therapists were employed at, reflecting their local context and when they transitioned from Platform 1 to Platform 2.

Individual characteristics were demographics (age, gender) and their professional background (educational background, advanced education in therapeutic approaches, preferences in therapeutic approach, years of experience with face-to-face therapy, and years of experience with iCBT). Data on contextual and individual characteristics were collected only the first time the therapists responded to the survey (either at T0 or T1).

### 2.6 Ethical considerations

The study was approved by the Data Protection Officer at Haukeland University Hospital and registered in the hospital's project registry (eProtokoll, project ID: 4696–4696).

### 2.7 Statistical analysis

The statistical analyses were conducted in IBM SPSS Statistics 30.0.0.0. Descriptive statistics (means, standard deviations, frequencies, and percentages) were calculated for age, gender, profession, theoretical preference, years of experience with face-to-face therapy, and years of experience with iCBT.

To answer our research question, linear mixed models (LMMs) were fitted to examine changes in TAM variables over time. Since the hospital trusts transitioned to the new platform at different times, the predictor, time, was operationalized as the number of months relative to each hospital trust's platform change. Time was centered on the month of transition, making the intercepts the expected values at baseline, i.e., the point in time when the hospital trusts changed platform. Negative values indicated assessments prior to the change, and positive values indicated assessments after the change. The resulting time variable ranged from −13 (13 months before the change) to +16 (16 months after the change) across hospital trusts.

To assess the change trajectories of each TAM variable, separate models were fitted for each outcome, resulting in 11 different models. To control the false discovery rate (FDR) across these multiple comparisons, we applied the Benjamini–Hochberg procedure to the set of p-values for the time slope coefficients (Benjamini and Hochberg, 1995). We report both unadjusted p-values and FDR-adjusted q-values, with statistical significance defined as q < 0.05.

For each outcome, we compared three linear mixed model specifications: (1) random intercept, (2) random intercept plus random slope, and (3) random intercept with hospital trust as an additional grouping level. Selection was based primarily on Bayesian Information Criterion (BIC), with Akaike's Information Criterion (AIC) considered when differences were minimal, favoring parsimony when fit was comparable. Model 1 was retained for most outcomes, while Model 3 was chosen for Behavioral Intention to use Platform 2, Perceived Usefulness for Therapists, and Voluntariness. Because we expected an initial reduction in acceptance levels followed by a potential recovery, the selected models were extended with a quadratic time term to account for nonlinear trajectories over time.

## 3 Results

### 3.1 Sample characteristics

The analytic sample comprised 46 of the 67 therapists (69%) delivering iCBT in the Norwegian specialist health care services, contributing a total of 146 observations. The sample contained therapists working at all 11 hospital trusts.

Sample characteristics are summarized in Table 2. Participants were predominantly female (69.6%), with a mean age of 42 years (SD = 10.48, range: 26 to 64). Most participants were clinical psychologists (39.1%) or specialist clinical psychologists (similar to board certification in other countries) (30.4%). Their clinical experience from face-to-face therapy varied, ranging from less than 1 year to 22 years (mean = 9.22, SD = 5.83), while their experience with iCBT ranged from less than a year to 9 years, averaging 2.3 years (SD = 1.8).

**Table 2:** Sample characteristics.

Variable	n	%	Mean	SD
Age			42	10.48

**Gender**				
Female	32	69.6%		
Male	13	28.3%		
Does not wish to disclose	1	2.2%		

**Profession**				
Clinical Psychologist	18	39.1%		
Specialist clinical psychologist	14	30.4%		
Nurse	1	2.2%		
Specialist nurse	6	13%		
Social Worker	2	4.4%		
Clinical social worker	2	4.4%		
Social educator	2	4.4%		
Clinical occupational therapist	1	2.2%		

**Years of Experience**				
With iCBT			2.27	1.67
With face-to-face therapy			9.22	5.83

**Preferred Therapy Other than CBT**				
Yes	17	37%		
No	29	63%		

### 3.2 Acceptance at baseline

Baseline acceptance for all outcomes is presented in Table 3. Apart from Behavioral Intention to Use Platform 1 (BI1) and Image, all variables had intercepts above 5 on the 7-point scale, indicating a moderate to high level of acceptance on most TAM constructs immediately before the platform change. In contrast, BI1 (4.44) indicates that the therapists' intention to use Platform 1 was only slightly positive. Image had the lowest baseline intercept (3.10), indicating that therapists tended to disagree that using iCBT enhanced their professional regard.

**Table 3:** Baseline intercepts and ICCs for all outcomes.

Outcome	Intercept (95% CI)	ICC therapist	ICC Trust
Behavioral intention to use Platform 1	4.44 [4.06, 4.82]	0.31	–
Behavioral intention to use Platform 2	5.15 [4.70, 5.61]	0.15	0.09
Perceived usefulness for themselves	6.07 [5.71, 6.43]	0.42	0.24
Perceived usefulness for their patients	5.98 [5.78, 6.17]	0.61	–
Perceived ease of use for themselves	5.92 [5.66, 6.18]	0.69	–
Perceived ease of use for their patients	5.56 [5.33, 5.79]	0.43	–
Subjective norm	5.80 [5.56, 6.03]	0.54	–
Image	3.10 [2.80, 3.40]	0.52	–
Voluntariness	5.09 [4.58, 5.60]	0.34	0.20
Perceptions of external control	6.19 [6.01, 6.37]	0.26	–
Training	6.19 [5.96, 6.42]	0.31	–

*Note*. ICC Therapist = proportion of variance at participant level; ICC Trust = proportion at hospital trust level.

### 3.3 Changes in acceptance over time

**Fig. 2:**
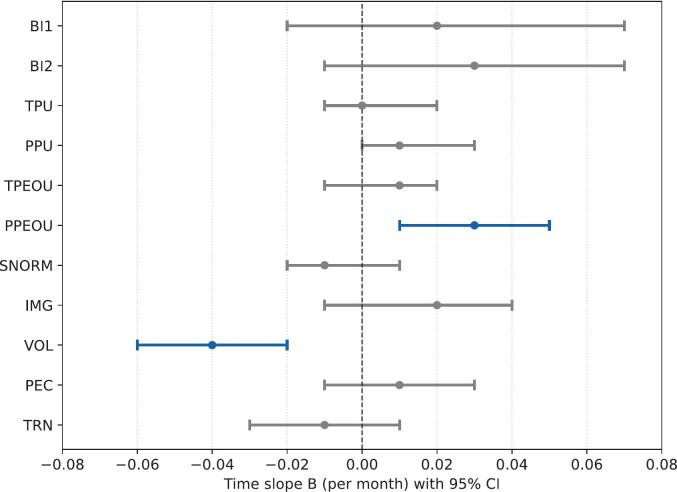
Forrest plot of time effects across outcomes. Forrest plot of time effects across outcomes. Points show estimated time slope (B per month) with 95% confidence intervals for each outcome. Color indicates FDR significance (q < 0.05): blue = significant, gray = non-significant. BI1 = Behavioral intention to use Platform 1, BI2 Behavioral intention to use Platform 2. TPU = Perceived usefulness for therapists, PPU = Perceived usefulness for patients, TPEOU = Perceived ease of use for therapists, PPEOU = Perceived ease of use for patients, SNORM = Subjective Norm, IMG = Image, VOL = Voluntariness, PEC = Perceptions of external control, TRN = Training.

Changes in TAM constructs over time are presented in Fig. 2 and Table 4. Three TAM variables showed statistically significant changes over time based on unadjusted p-values: Perceived Usefulness for Patients (PPU; B = 0.01, p = 0.046), Perceived Ease of Use for Patients (PPEOU; B = 0.03, p = 0.005) and Voluntariness (VOL; B = −0.04, p = 0.002), but after controlling for multiple comparisons (FDR) only PPEOU (q = 0.028) and VOL (q = 0.022) remained significant.

**Table 4:** Fixed effect of time with FDR-adjusted q-values.

Outcome	Slope B	95% CI	df	t	p / FDR q
BI1	0.02	[−0.02, 0.07]	100.25	1.08	p = 0.283; q = 0.429
BI2	0.03	[−0.01, 0.07]	78.74	1.34	p = 0.185; q = 0.407
TPU	0.00	[−0.01, 0.02]	109.29	0.41	p = 0.681; q = 0.681
PPU	0.01	[0.00, 0.03]	114.93	2.02	p = 0.046; q = 0.169
TPEOU	0.01	[−0.01, 0.02]	107.15	1.02	p = 0.312; q = 0.429
PPEOU	0.03⁎	[0.01, 0.05]	128.26	2.88	p = 0.005; q = 0.028
SNORM	−0.01	[−0.02, 0.01]	121.22	−0.81	p = 0.418; q = 0.477
IMG	0.02	[−0.01, 0.04]	125.74	1.43	p = 0.156; q = 0.407
VOL	−0.04⁎	[−0.06, −0.02]	116.58	−3.19	p = 0.002; q = 0.022
PEC	0.01	[−0.01, 0.03]	139.56	1.03	p = 0.306; q = 0.429
TRN	−0.01	[−0.03, 0.01]	137.14	−0.78	p = 0.434; q = 0.477

*Note.* BI1 = Behavioral intention to use Platform 1; BI2 = Behavioral intention to use Platform 2; TPU = Perceived usefulness for therapists; PPU = Perceived usefulness for patients; TPEOU = Perceived ease of use for therapists; PPEOU = Perceived ease of use for patients; SNORM = Subjective norm; IMG = Image; VOL = Voluntariness; PEC = Perceptions of external control; TRN = Training. Positive B = increase per month since platform change; negative B = decrease. q-values are Benjamini–Hochberg (FDR) adjusted across the 11 constructs.

⁎ <0.05.

**Fig. 3:**
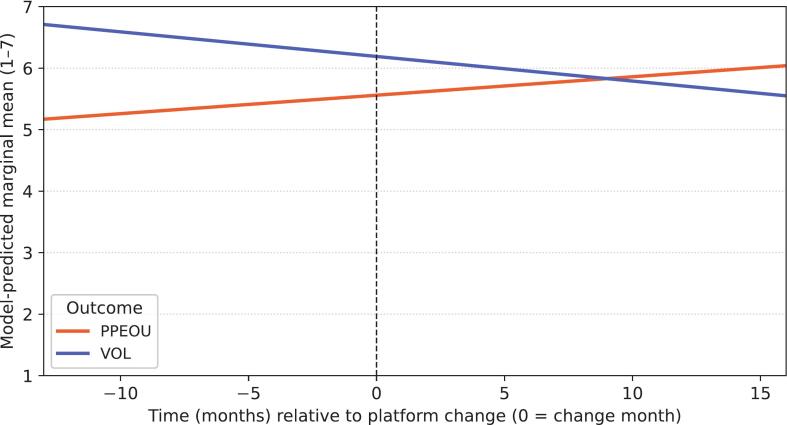
Trajectories predicted marginal means for PPEOU and VOL over time. Trajectories for Perceived ease of use for patients (PEOU), and voluntariness (VOL) over time. Lines show fixed-effect predicted marginal means over months relative to the platform change (0 = change month). Predictions use the exact intercepts and slopes: PPEOU (intercept: 5.56, slope: +0.03), VOL (intercept: 5.09, slope: −0.04).

PPEOU increased significantly over time, indicating that the therapists' perceptions of how easy iCBT was for their patients to use improved during the transition to the new platform. In contrast, VOL decreased significantly over time, indicating that therapists perceived their use of iCBT as less voluntary during the transition. The trajectory of predicted marginal means for these outcomes is presented in Fig. 3. The remaining eight TAM variables did not show significant change over time, indicating stable perceptions across the transition period for these constructs.

Significant quadratic time effects were observed for Subjective Norm (SNORM; p = 0.049), Perceptions of External Control (PEC; p = 0.036) and Training (TRN; p < 0.001). The clearest curvilinear pattern was observed for Training, reflecting an initial decrease followed by recovery over time.

### 3.4 Variability across individuals and hospital trusts

Detailed variance components for random effects are presented in Appendix B. Residual variance was significant in all models (ps < 0.001), indicating that therapists' scores varied substantially over time. At baseline, therapists differed systematically from each other on all outcomes except Behavioral Intention to use Platform 2 (BI2), where the participant-level intercept was not significant (p = 0.246). Participant-level ICCs ranged from 0.15 (BI2) to 0.69 (TPEOU), showing that individual differences explained 15% to 69% of the variance, with particularly high clustering for TPEOU (0.69) and PPU (0.61).

Hospital trust–level random intercepts were not statistically significant for any outcome (all ps > 0.05). Although descriptive ICCs indicated that a small to moderate proportion of variance was located at the organizational level for some constructs, the limited number of hospital trusts and uneven cluster sizes preclude firm conclusions regarding organizational effects.

## 4 Discussion

This study examined how therapists' acceptance of iCBT changed during a national transition to a new iCBT platform, with particular attention to the dual-user nature of iCBT. Overall, therapists' acceptance of iCBT was largely stable across the transition, with significant changes observed only for perceptions of ease of use for patients and voluntariness. Furthermore, Training showed an initial reduction followed by recovery over time. Prior to the transition, therapists reported high acceptance across most TAM constructs, alongside lower Image scores and only slightly positive behavioral intention to continue using the outgoing platform (Platform 1). The data also revealed large individual differences in acceptance on all TAM variables except their behavioral intention to use the new platform (Platform 2). Descriptive ICCs indicated that most variance occurred between therapists, with smaller proportions attributable to differences between hospital trusts. However, none of the tests for random effects at the hospital trust level were significant. The following discussion reflects on what these findings indicate for the implementation of iCBT and the acceptance and use of dual-user technologies.

### 4.1 High and stable acceptance of iCBT

The finding that acceptance of iCBT was high at the outset and remained stable throughout the platform transition indicates two points. Firstly, the high acceptance in our sample suggests that iCBT can be an acceptable treatment approach for a variety of clinicians within established iCBT services. Secondly, the stability throughout the transition period suggests that this high level of acceptance is resilient to disruption among experienced iCBT therapists. In our results, this resilience also persisted while Image remained low, suggesting that concerns about prestige or status are not decisive factors in the use of these treatments.

While most constructs remained stable over time, Voluntariness and Perceived Ease of Use for patients showed changes. However, the scale used to measure Voluntariness showed low internal consistency at baseline (α = 0.44), making it unclear whether the results reflect a true change or measurement instability. Training was the only outcome that displayed a curvilinear trajectory in line with our expectations, with an initial decrease followed by recovery. This suggests that therapists initially perceived their training as insufficient for using the new platform, before this decrease recovered over time. However, this did not affect other acceptance measures. One possible explanation is that experienced therapists view iCBT as one coherent approach rather than a collection of components (i.e. content, technology, messaging system). Although therapists perceived a need for more training in the new platform, this did not alter their overall understanding of iCBT as a coherent approach, and their acceptance of iCBT remained stable.

From an implementation standpoint, these findings suggest that while establishing iCBT in clinical practice is a challenge, once it is established, therapists' acceptance is resilient to platform changes. Furthermore, previous research on Norwegian iCBT therapists showed that the highest levels of acceptance were achieved through a structured top-down implementation process in which therapists with diverse professional backgrounds and characteristics were mandated to use iCBT while receiving supervision and support (Nævdal et al., 2025). This suggests that the primary barrier to scaling up iCBT in the Norwegian context may not be therapists' willingness to adopt the treatment, but rather their lack of experience with it. While several efforts to disseminate iCBT have been conducted in Norway, hands-on experience with the treatments is only available to therapists delivering iCBT. This means that only 67 therapists have gained such experience, while there are an estimated 10,265 clinical psychologists in the Norwegian specialist health care services (Statistics Norway, 2024). To scale up the use of iCBT, strategies that provide structured exposure, such as temporary mandated use combined with supervision and support, may therefore be beneficial.

### 4.2 Individual and organizational differences

Although overall acceptance was high and stable, we also found substantial variance in acceptance between individual therapists at baseline. This variability seems reasonable and is consistent with the Technology Acceptance Model, which emphasizes individual differences in perceptions and attitudes. With this in mind, technology transitions may benefit from flexible onboarding and support where efforts are adaptable to individual needs, rather than a more rigid strategy.

More surprising was the lack of significant differences between hospital trusts. Previous research highlights that local context often influences implementation and that tailoring strategies to each setting can improve outcomes (Baker et al., 2015; Vis et al., 2023). While our findings may be due to statistical limitations in our sample, the transition to the new platform was also coordinated as a national strategy, supported by established cooperative networks and standardized procedures across the hospital trusts. These types of centralized approaches have previously been shown to reduce variability and promote cohesive implementation across sites (Long et al., 2024).

While individual therapists varied on most acceptance outcomes, one variable did not show significant individual differences. This was their behavioral intention toward Platform 2 (BI2). At baseline, therapists had no practical experience with Platform 2, meaning their responses reflected expectations rather than hands-on use. This finding is consistent with TAM, in which acceptance is shaped by users' perceptions of a technology rather than by the technology itself. In the absence of hands-on experience, the relatively high and uniform BI2 scores likely reflect shared expectations about the new platform, rather than evaluations based on individual use. As therapists gain experience, individual differences in BI2 scores may emerge, as their evaluations become informed by personal needs and contextual factors.

From an implementation perspective, this suggests that pre-implementation communication and organizational framing may play an important role in shaping early acceptance of a treatment platform and facilitating alignment across sites. However, because no standardized communication strategy was implemented or evaluated in this study, it is not possible to determine specific factors that may have contributed to the high BI2 scores at baseline.

### 4.3 Dual-user perspective on acceptance

The transition to a new platform was associated with a significant improvement in therapists' perceived ease of use for their patients (PPEOU), but not in their perceptions of their own ease of use (TPEOU) or usefulness (TPU). This suggests that when evaluating iCBT, the therapists differentiate between their own and their patients' experiences. Furthermore, therapists' behavioral intention to use Platform 2 (BI2) was also higher than their intention to use Platform 1 (BI1). Although the study does not determine whether this increase in behavioral intention is attributable to their belief that the new platform would be easier to use for patients, this may indicate that therapists' behavioral intention, and in turn user behavior, is partially contingent on their perceptions of their patients' experiences. If this is the case, ensuring therapists perceive iCBT as useful and easy to use for their patients may promote adoption and sustained engagement. Future research should adapt technology acceptance models for multi-user contexts and investigate these relationships more explicitly.

### 4.4 Limitations and strengths

Several limitations should be considered when interpreting these findings. Firstly, the sample is modest and drawn from a specific national context among experienced iCBT therapists. While the LMMs allowed us to examine variance across individuals and organizations, the modest sample size and small number of hospital trusts reduce the study's statistical power to detect all effects of the transition to a new platform – particularly at the hospital trust level. Although the specific context may limit generalizability to other countries or settings where iCBT is newly introduced, the sample represents 69% of the total population of iCBT therapists in Norway. This strengthens the internal validity and suggests that the results are a reasonable representation of the Norwegian population of iCBT therapists.

Secondly, the study relied on self-report data and did not measure therapist user behavior or any patient outcomes. The findings may therefore be subject to known self-report biases, and the adaptation/translation of the TAM-variables may have affected their validity and reliability. While these limitations should be acknowledged, the use of self-report measures and adapted scales is consistent with prior research on technology acceptance (Perski and Short, 2021; Rahimi et al., 2018; Venkatesh and Bala, 2008; Venkatesh and Davis, 2000). Internal consistency was also acceptable to excellent for most scales, but the lower scores on Voluntariness and Subjective Norm indicate that these scales may have reduced sensitivity to detect change.

A key strength of this study is its naturalistic design, examining acceptance trajectories during a real-world national platform transition embedded in routine care using a representative sample of Norway's iCBT-therapists. The longitudinal design allowed us to examine how acceptance evolved during the transition, rather than relying on cross-sectional snapshots. Modeling time as a continuous variable enabled us to accommodate variation in transition timing across hospital trusts and to capture potential gradual changes in acceptance. Lastly, by explicitly examining therapists' perceptions of patient usability, this study addresses a rarely examined but theoretically important aspect of acceptance in guided digital interventions.

### 4.5 Conclusion and practical implications

The present findings suggest that high and stable acceptance of iCBT is achievable among clinicians once practical experience with iCBT has been established. Once established, platform transitions do not necessarily undermine core acceptance in mature services. High behavioral intention to use a new platform can also be achieved before therapists have gained hands-on experience, indicating that their acceptance can be based on expectations and is not dependent on the objective functionality or the platform's user interface. During platform transitions, interventions targeting therapists' expectations of the new platform may therefore facilitate a smooth transition by promoting early acceptance. Furthermore, our results highlight that therapists differentiate between their own and their patients' user experience. Since therapists hold a key role in the utilization of iCBT, it is likely that how they perceive their patients' user experiences with iCBT is important for adoption and sustained use.

iCBTs are complex in nature, and just as design choices may affect which scientific inferences can be drawn about their effectiveness (Goldberg et al., 2023), therapists' prior experience, expectations and understanding of their patients' user experiences can interact with evaluation designs and influence both outcome measures and their interpretations. Addressing these factors explicitly in future study designs can help align theoretical models of acceptance more closely with experimental approaches for assessing digital interventions.

## List of abbreviations

iCBT: Therapist-guided internet-delivered cognitive behavioral therapy
TAM: The Technology Acceptance Model
BI: Behavioral intention
PU: Perceived usefulness
PEOU: Perceived ease of use
BI1: Behavioral intention to use Platform 1
BI2: Behavioral intention to use Platform 2
TPU: Perceived usefulness for the therapist
PPU: Perceived usefulness for the patient
TPEOU: Perceived ease of use for the therapist
PPEOU: Perceived ease of use for the patient
SNORM: Subjective Norm
IMG: Image
VOL: Voluntariness
PEC: Perceptions of External Control
TRN: Training

## CRediT authorship contribution statement

The conceptualization of the study was done by RN, RK and CV, and data collection was conducted by RN and RK. Data analyses were conceptualized by RN and DB and conducted by RN. All data interpretation and drafting of the manuscript was done by RN. All authors critically reviewed and revised the manuscript. The final version was read and approved by all authors.

## Disclaimer

The authors are alone responsible for the content in this article. These do not necessarily reflect the policies or positions of the funding bodies involved.

## Role of sponsor

The funding body played no role in the design of the study, the collection, analysis, and interpretation of data or in writing the manuscript.

## Declaration of Generative AI and AI-assisted technologies in the writing process

During the preparation of this work the author(s) used Microsoft Copilot, a large language model powered by OpenAI technology, to improve readability, to identify and remove redundant or unnecessarily lengthy text, and to create tables. After using Copilot, the author(s) reviewed and edited the content as needed and take(s) full responsibility for the content of the published article.

## Funding

The 10.13039/501100005416Research Council of Norway, Grant number: 309264.

## Declaration of competing interest

The authors declare that they have no known competing financial interests or personal relationships that could have appeared to influence the work reported in this paper.

## Figures and Tables

**Table:** 

TAM-variable	Original items and source	Norwegian items
Behavioral intention to use Platform 1	1. Assuming I had access to the system, I intend to use it 2. Given that I had access to the system, I predict that I would use it 3. I plan to use the system in the next <n > months	Om jeg *bare* hadde tilgang til behandlingen slik den er i PLATFORM 1, ville jeg benyttet den til å behandle alle pasienter med … 1. … depresjon som hovedvanske 2. … sosial angst som hovedvanske 3. … panikklidelse som hovedvanske
Behavioral intention to use Platform 2		Om jeg *bare* hadde tilgang til behandlingen slik den er i PLATFORM 2, ville jeg benyttet den til å behandle alle pasienter med … 1. … depresjon som hovedvanske 2. … sosial angst som hovedvanske 3. … panikklidelse som hovedvanske
Perceived usefulness for themselves	1. Using the system improves my performance in my job 2. Using the system in my job increases my productivity 3. Using the system enhances my effectiveness in my job 4. I find the system to be useful in my job	Med eBehandling kan jeg gi god behandling for … 1. … depresjon 2. … sosial angst 3. … panikklidelse 4. Muligheten til å bruke eBehandling i arbeidet mitt bidrar til at jeg er tilfreds på jobben 5. Med eBehandling kan jeg gjøre jobben min på en effektiv mate 6. eBehandling er nyttig for meg i jobben min
Perceived usefulness for their patients		eBehandling er et godt behandlingstilbud for pasienter jeg møter som har … 1. … depresjon 2. … sosial angst 3. … panikklidelse 4. Å bruke eBehandling gir pasientene mine fordeler som de ikke har uten eBehandling 5. eBehandling er nyttig for mine pasienter
Perceived ease of use for themselves	1. My interaction with the system is clear and understandable 2. Interacting with the system does not require a lot of my mental effort 3. I find the system to be easy to use 4. I find it easy to get the system to do what I want it to do	1. Det er enkelt for meg å bruke eBehandling i jobben min generelt Det er enkelt for meg å bruke eBehandling til å behandle … 2. … pasienter med depresjon 3. … pasienter med sosial angst 4. … pasienter som har panikklidelse
Perceived ease of use for their patients		1. Det er enkelt for mine pasienter å bruke eBehandling Å motta behandling gjennom eBehandling er enkelt for pasientene mine som har … 2. … depresjon 3. … sosial angst 4. … panikklidelse
Subjective norm	1. People who influence my behavior think that I should use the system. 2. People who are important to me think that I should use the system. 3. The senior management of this business has been helpful in the use of the system. 4. In general, the organization has supported the use of the system	1. Mennesker som er viktige for meg er positive til at eBehandling brukes til å behandle pasienter 2. Mine overordnede på jobben er positive til at eBehandling brukes til å behandle pasienter 3. Arbeidsplassen min generelt er positiv til eBehandling
Image	1. People in my organization who use the system have more prestige than those who do not 2. People in my organization who use the system have a high profile 3. Having the system is a status symbol in my organization	1. På min arbeidsplass anses erfaring med eBehandling som et tegn på høy kompetanse 2. Ansatte på min arbeidsplass som bruker eBehandling har høyere anseelse enn ansatte som ikke bruker eBehandling 3. Å bruke eBehandling er et statussymbol på min arbeidsplass
Voluntariness	1. My use of the system is voluntary 2. My supervisor does not require me to use the system 3. Although it might be helpful, using the system is certainly not compulsory in my job	1. Det er frivillig for meg å benytte eBehandling i arbeidet mitt 2. Det er mitt valg om jeg skal ta i bruk eBehandling til å behandle en spesifikk pasient 3. Jeg opplever ikke press om å bruke eBehandling i jobben min
Perceptions of external control	1. I have control over using the system 2. I have the resources necessary to use the system 3. Given the resources, opportunities and knowledge it takes to use the system, it would be easy for me to use the system 4. The system is not compatible with other systems I use	1. Jeg har støtten jeg trenger for å levere god behandling gjennom eBehandling 2. Om jeg har tekniske vansker i eBehandling, finnes det tilgjengelig støtte som kan hjelpe meg 3. Om jeg møter kliniske utfordringer mens jeg bruker eBehandling, finnes det tilgjengelig støtte som kan hjelpe meg 4. Jeg mottar tilstrekkelig veiledning til å gi god behandling i eBehandling 5. eBehandling fungerer godt sammen med resten av. arbeidsplassen min (rutiner, organisering eller lignende)
Training	1. Your level of training for the use of TECHNOLOGY was appropriate prior to the implementation. 2. Currently, your level of training for the use of TECHNOLOGY is appropriate. 3. You have gained a complete understanding of the features, functions, and abilities of TECHNOLOGY 4. More training of TECHNOLOGY would help the implementation process.	Jeg har mottatt opplæringen jeg trenger for å bruke … 1. … eBehandlingsplatformen 2. … behandlingen for depresjon 3. … behandlingen for sosial angst 4. … behandlingen for panikklidelse

*Note.* The original items for the scales regarding Behavioral Intention, Perceived Usefulness and Perceived ease of use, were translated and adapted from Davis (1989) and Davis et al. (1989). All scales measuring the antecedent variables except Image and Training were translated and adapted from an overview of psychometrically validated items and scales published by Venkatesh and Bala (2008). Image was translated and adapted from Moore and Benbasat (1991), and Training from Bradley and Lee (2007).

The translation and development of the Norwegian items are described in Nævdal et al. (2025).

**Table:** 

Outcome	Parameter	Estimate	SE	Z	p	95% CI
BI1	Residual	1.356	0.236	5.74	<0.001	[0.963, 1.908]
BI1	Intercept (HT)	–	–	–	–	–
BI1	Intercept (ID)	0.611	0.289	2.12	0.034	[0.242, 1.542]
BI2	Residual	1.306	0.226	5.78	<0.001	[0.931, 1.834]
BI2	Intercept (HT)	0.156	0.178	0.87	0.382	[0.017, 1.468]
BI2	Intercept (ID)	0.260	0.224	1.16	0.246	[0.048, 1.406]
TPU	Residual	0.242	0.035	6.94	<0.001	[0.183, 0.322]
TPU	Intercept (HT)	0.172	0.124	1.39	0.165	[0.042, 0.706]
TPU	Intercept (ID)	0.305	0.100	3.06	0.002	[0.161, 0.579]
PPU	Residual	0.211	0.031	6.89	<0.001	[0.159, 0.281]
PPU	Intercept (HT)	–	–	–	–	–
PPU	Intercept (ID)	0.334	0.094	3.56	<0.001	[0.193, 0.580]
TPEOU	Residual	0.284	0.042	6.74	<0.001	[0.213, 0.380]
TPEOU	Intercept (HT)	–	–	–	–	–
TPEOU	Intercept (ID)	0.631	0.175	3.60	<0.001	[0.366, 1.088]
PPEOU	Residual	0.520	0.074	7.04	<0.001	[0.394, 0.687]
PPEOU	Intercept (HT)	–	–	–	–	–
PPEOU	Intercept (ID)	0.399	0.129	3.09	0.002	[0.212, 0.753]
SNORM	Residual	0.390	0.056	7.01	<0.001	[0.295, 0.516]
SNORM	Intercept (HT)	–	–	–	–	–
SNORM	Intercept (ID)	0.466	0.134	3.48	<0.001	[0.265, 0.818]
IMG	Residual	0.683	0.096	7.12	<0.001	[0.519, 0.899]
IMG	Intercept (HT)	–	–	–	–	–
IMG	Intercept (ID)	0.742	0.209	3.55	<0.001	[0.427, 1.289]
VOL	Residual	0.756	0.108	7.01	<0.001	[0.572, 1.000]
VOL	Intercept (HT)	0.320	0.245	1.31	0.190	[0.072, 1.430]
VOL	Intercept (ID)	0.550	0.197	2.79	0.005	[0.272, 1.110]
PEC	Residual	0.524	0.072	7.25	<0.001	[0.399, 0.686]
PEC	Intercept (HT)	–	–	–	–	–
PEC	Intercept (ID)	0.180	0.075	2.40	0.017	[0.079, 0.407]
TRN	Residual	0.728	0.100	7.24	<0.001	[0.555, 0.954]
TRN	Intercept (HT)	–	–	–	–	–
TRN	Intercept (ID)	0.323	0.119	2.71	0.007	[0.157, 0.666]

*Note*. SE = standard error; Z = Wald Z statistic; CI = Confidence Interval; HT = hospital trust Level; ID = Participant Level; BI1 = Behavioral intention to use Platform 1; BI2 = Behavioral intention to use Platform 2; TPU = Perceived usefulness for therapists; PPU = Perceived usefulness for patients; TPEOU = Perceived ease of use for therapists; PPEOU = Perceived ease of use for patients; SNORM = Subjective norm; IMG = Image; VOL = Voluntariness; PEC = Perceptions of external control; TRN = Training.
